# Enhancing Dark Chocolate with Fermented Laver (*Porphyra umbilicalis*): Effects on Sensory Characteristics and Consumer Acceptance

**DOI:** 10.3390/foods15122047

**Published:** 2026-06-06

**Authors:** Zach Adams, Matthew B. McSweeney

**Affiliations:** School of Nutrition and Dietetics, Acadia University, Wolfville, NS B4P 2R6, Canada; 0210969a@acadiau.ca

**Keywords:** macroalgae, seaweed, fermentation, sensory evaluation, chocolate, consumer, acceptability, check-all-that-apply

## Abstract

Seaweed is increasingly recognized as a sustainable food source with the potential to contribute to Western diets; however, consumer acceptance remains low. This is partly due to undesirable sensory attributes, including fishy and marine flavours present when seaweed is incorporated into foods. Fermentation has been proposed to minimize these off-flavours. This study evaluated the impact of fermentation on the sensory properties of *Porphyra umbilicalis* (laver). Dark chocolate (54.5% cocoa) was enriched with laver fermented using three cultures: *Streptococcus thermophilus* (ST), *Pediococcus pentosaceus* (PP), and *Aspergillus oryzae* (AO). Five formulations were assessed for consumer acceptability (*n* = 83) using nine-point hedonic scales and check-all-that-apply (CATA) questionnaires for sensory and emotional responses. All seaweed-containing samples received significantly lower liking scores than the control (overall liking: control = 7.8 ± 0.9; seaweed samples ranging from 5.7 ± 1.8 to 6.6 ± 1.5). Among seaweed-containing samples, AO-fermented laver achieved the highest flavour liking (6.5 ± 1.7) and overall liking (6.6 ± 1.5) scores, both significantly higher than the unfermented sample (flavour: 5.7 ± 1.8; overall: 5.7 ± 1.8; *p* < 0.05). Correspondence analysis of CATA data explained 88.01% of total variation, with the control associated with positive sensory attributes (sweet, creamy, smooth) while seaweed samples were characterized by off-flavours, fishy, and earthy descriptors. Emotional profiling using the EsSense25 profile indicated that the unfermented sample was exclusively associated with negative emotions (bored, worried, disgusted), while AO was associated with seven positive emotional terms. These findings suggest that fermentation, particularly with AO, may improve the sensory acceptability of seaweed in Western food products when incorporated into familiar matrices. Further research should examine different seaweed species and fermentation strategies to enhance consumer acceptance.

## 1. Introduction

The global population is projected to reach 9.7 billion by 2050, requiring an estimated 70% increase in food production to meet rising demand [1]. This challenge has intensified interest in sustainable and alternative food sources that can alleviate pressure on conventional agricultural systems. Seaweed has emerged as a promising candidate in the food industry due to its high productivity and minimal resource requirements. However, modern seaweed production remains highly concentrated, with approximately 99% of global cultivated supply originating from East Asia [2]. Although global markets for whole seaweed are expanding, this growth is largely driven by the diffusion of traditional Asian cuisines, with limited innovation in novel food applications in the Western world. Consequently, despite its recognized sustainability advantages, including cultivation without arable land, freshwater, or fertilizers, seaweed consumption remains low in Western diets [3].

Seaweed, or macroalgae, encompasses diverse freshwater and marine protists classified into brown (*Phaeophyceae*), green (*Chlorophyceae*), and red (*Rhodophyceae*) groups based on pigmentation [4]. While composition varies by species, seaweeds are generally rich in fibre and protein, with low lipid content [5]. They are also recognized as functional foods due to their high micronutrient levels and associated health benefits, including antioxidant and antimutagenic activities [6,7]. Commercially, seaweeds are currently processed into widely used food ingredients such as carrageenan and alginate [8].

Despite these advantages, sensory challenges remain a key barrier to broader acceptance when seaweeds are incorporated as a whole ingredient. Seaweeds contribute both desirable and undesirable flavour attributes when incorporated into foods. Their high concentrations of glutamic and aspartic acids impart umami characteristics, supporting their traditional use in savoury products such as soups and baked goods [9]. However, Western consumers often perceive seaweed as having fishy or marine-like flavours and odours, which are generally considered undesirable [10]. Consumer acceptability remains a major barrier to the widespread adoption of whole seaweeds and seaweed-based products. Sensory evaluation studies have shown that seaweed-enriched foods are often poorly received, with participants describing them using negative attributes such as oily, slimy, chunky, and fish-like [11]. In addition, seaweed is frequently considered underutilized due to food neophobia, defined as the reluctance to consume unfamiliar foods [12]. Despite sustainability and nutritional advantages, the distinctive flavour and texture profiles of seaweeds remain significant barriers to their acceptance among Western consumers [9] and seaweed-enriched products are frequently rated as less palatable than their non-enriched counterparts [13,14]. To increase adoption among Western consumers, continued efforts are needed to develop innovative seaweed-based products that incorporate familiar sensory characteristics and product formats.

Fermentation represents a promising yet underexplored approach for improving the sensory properties of seaweed. Microbial fermentation can modify flavour by reducing undesirable volatile compounds and generating new aroma-active metabolites [15]. Commonly studied cultures include lactic acid bacteria, *Aspergillus oryzae*, and *Saccharomyces cerevisiae* [15,16,17,18]. Several studies have investigated the effects of fermenting seaweeds or incorporating them into food products to improve sensory quality [10,15,16,19]. Fermentation with *Saccharomyces cerevisiae* has been shown to reduce or eliminate volatile organic compounds (VOCs) associated with fishy, pungent, and rancid aromas, including hexanal, 1-octen-3-ol, and 2,4-decadienal, while introducing desirable floral, sweet, citrusy, and fruity notes [15,16]. Similarly, fermentation of *Laminaria japonica* with *Aspergillus oryzae* significantly reduced key odor-active compounds, including isovaleric acid and octanal, which contributed substantially to the overall odour profile of unfermented samples [10]. The effectiveness of fermentation depends on the microbial strain used, with *S. cerevisiae* and *Bacillus subtilis* generally producing more favourable sensory outcomes than lactic acid bacteria [15]. Collectively, these findings demonstrate that fermentation can enhance the sensory properties of seaweed by producing acceptable flavours.

While analytical techniques such as gas chromatography–mass spectrometry (GC–MS) have been widely used to characterize fermentation-induced changes in seaweed volatiles, relatively few studies have examined consumer perception of these modifications. Instrumental analyses provide valuable chemical insights but do not fully capture consumer acceptance or preference. Integrating consumer-based sensory methods, including hedonic scales and check-all-that-apply (CATA) questionnaires, enables a more comprehensive evaluation of product acceptability. The check-all-that-apply (CATA) method, particularly when combined with hedonic scaling, offers a valuable approach for the sensory evaluation of novel food products [20]. CATA enables untrained consumers to simultaneously select multiple sensory descriptors from a predefined list, generating rapid and thorough data on perceived attributes such as flavour, aroma, texture, and appearance [21]. Furthermore, the integration of CATA with hedonic ratings allows for sensory characteristics to be directly linked to consumer preferences. This combined approach is particularly relevant for products containing unfamiliar ingredients, such as seaweed, as it facilitates the identification of attributes that drive acceptance or rejection and supports targeted formulation strategies to enhance overall consumer appeal [22]. In addition, assessing emotional responses to food provides a more complete understanding of consumer perception and product experience [23]. While hedonic ratings quantify overall liking, they do not capture the affective factors that influence consumer behaviour, such as feelings of comfort, excitement, nostalgia, or disgust. The EsSense25 Profile, a validated lexicon of 25 emotion terms, was developed to systematically measure these responses in food-related consumer research [24]. Incorporating the EsSense25 Profile alongside traditional hedonic scales enables the identification of emotions elicited by a product and facilitates examination of how these emotional responses relate to acceptance, purchase intent, and product positioning.

The objective of this study was to assess consumer perception of dark chocolate made with *Porphyra umbilicalis* (laver), grown off the Atlantic coast, following fermentation with *Streptococcus thermophilus*, *Pediococcus pentosaceus*, and *Aspergillus oryzae*. Fermented seaweed samples were incorporated into dark chocolate, a widely accepted product, to determine whether integration into a familiar food matrix could enhance consumer acceptance. Chocolate is a suitable candidate for seaweed incorporation due to its complex flavor profile, which can help balance the marine and umami notes of seaweed [25,26].

## 2. Materials and Methods

### 2.1. Samples

Samples were prepared using N° 811 (54.5%) dark chocolate Callets™ (Callebaut, Wieze, Belgium), with individual portions weighing approximately 3 g. Dried whole-leaf laver (*Porphyra umbilicalis*) was obtained from Maine Coast Sea Vegetables (Hancock, ME, USA). *Aspergillus oryzae* spores were sourced from Angel Yeast Co. (Yichang, China), while *Pediococcus pentosaceus* and *Streptococcus thermophilus* were obtained from BulkProbiotics (Marietta, GA, USA). Samples are hereafter identified by fermentation culture (e.g., *S. thermophilus* = ST). These fermentation cultures were chosen based on preliminary trials by the researchers and a review of the literature [15,16,17,18].

Seaweed was prepared by rehydrating 30 g of dried laver in demineralized water for 20 min, draining, and combining with additional demineralized water to a final mass of 1030 g. The mixture was boiled for 20 min, then cooled to <30.0 °C. A 1% (*w*/*w*) inoculum (10 g) of the respective culture was added, and samples were mixed, transferred to glass reagent bottles, and fermented at 30.0 °C for 72 h in a water bath (Boekel Scientific, Feasterville, PA, USA). Following fermentation, samples were dehydrated at 52.0 °C for 20 h in a Excalibur 3926TB Dehydrator (Sacramento, CA, USA), ground using a mortar and pestle, and sieved (130 µm). An unfermented control underwent identical preparation without inoculation. Powders were stored at 4.0 °C until use. Five individual fermentation batches were prepared for each strain, pooled to create a homogeneous seaweed powder, and subsequently used to produce three replicate batches of chocolate for each formulation (outlined below).

Chocolate samples (Unfermented, ST, AO, PP), as well as control samples (without seaweed addition), were prepared three days prior to the sensory trial. Chocolate, in 2000 g batches, was heated to 48.0 °C in a double boiler, cooled to 29.0 °C, and then placed in the water bath and reheated to 32.0 °C. Once the chocolate had reached this temperature, seaweed powder was incorporated as an additive at 6% *w*/*w* without replacing any existing ingredient in the formulation, or the chocolate was left unmodified as a control (no seaweed powder addition). An incoporation level of 6% *w*/*w* was chosen based on preliminary evaluations by research assistants experienced in food product development and employed by the Centre for the Sensory Research of Food (CSRF) (Acadia University, Wolfville, NS, Canada). The 6% *w*/*w* incorporation level was selected to align with the upper range of seaweed addition levels reported in the literature for chocolate-type products (1–7.5% *w*/*w*) [25,26]. Samples were molded, set under refrigeration, portioned (3 g), coded, and stored at refrigeration temperature until evaluation. Each sample was placed in a 2 oz container, sealed with a lid, labelled with a randomized three-digit code, and stored in a refrigerator (4.0 °C) until evaluation. Sample presentation followed the procedure by Macfie [27].

### 2.2. Participants

Participants were recruited from the Annapolis Valley, Nova Scotia, Canada, through email outreach and word of mouth. Eligibility screening included assessment of food allergies, regular chocolate consumption, and willingness to consume seaweed. A total of 83 participants were enrolled in the study, of whom 73% identified as female and 27% as male. The mean age of participants was 43.4 ± 20.2 years.

### 2.3. Sensory Evaluation

Participants were received in a pre-screening area at the CSRF, where they provided written informed consent prior to participation. They were then seated in individual sensory booths and evalauted the samples using a questionnaire (Supplementary Material S1) presented by Compusense software ( Version 26.0.36796, Guelph, ON, Canada) on an iPad. Each sample was evaluated for appearance, flavour, texture, and overall liking using a nine-point hedonic scale (1 = “Dislike extremely”, 5 = “Neither like nor dislike”, 9 = “Like extremely”). Following each evaluation, participants completed a CATA question to indicate perceived sensory attributes. The CATA list comprised 24 attributes (aftertaste, astringent, bitter, bland, chewy, creamy, crumbly, earthy, fishy, grainy, gritty, hard, herbal, mouthcoating, nutty, off-flavour, rough, salty, sandy, savoury, smooth, soft, sour, and sweet), selected based on the literature describing the sensory characteristics of chocolate and seaweed [26,28,29,30,31,32,33,34,35,36,37]. Participants also completed a second CATA question assessing emotional responses using the EsSense25 Profile (active, adventurous, aggressive, bored, calm, disgusted, enthusiastic, free, good, good-natured, guilty, happy, interested, joyful, loving, mild, nostalgic, pleasant, satisfied, secure, tame, understanding, warm, wild, worried) [25]. After evaluating all samples, participants were invited to provide additional comments and complete an exit questionnaire on seaweed perceptions (Table 1) and demographic information.

### 2.4. Statistical Analysis

Hedonic scale data were analyzed using a two-way analysis of variance (ANOVA), with sample treated as a fixed factor and participant as a random factor. Significant differences among samples were further examined using Tukey’s Honestly Significant Difference (HSD) test. CATA data for the sensory properties were analyzed following the procedure described by Meyners et al. [38]. The frequency of each attribute selected for each sample was calculated, and differences among samples were assessed using Cochran’s Q test. Correspondence analysis was performed to visualize relationships between samples and sensory attributes. Penalty-lift analysis was conducted by combining overall liking scores with CATA frequencies to determine the impact of specific attributes on consumer acceptance. The same analytical approach was applied to emotional response data collected using the EsSense25 CATA [25]. Descriptive statistics were used to summarize responses to seaweed consumption questions. All analyses were conducted using XLSTAT (Version 2025.1, Lumivero, Denver, CO, USA) within Microsoft Excel, with statistical significance set at *p* < 0.05.

## 3. Results and Discussion

### 3.1. Participants

Participant responses to the belief questions (Table 1) indicate generally positive attitudes toward seaweed as a food ingredient, particularly regarding its perceived health benefits and sensory appeal. Previous research has consistently shown that consumers in Western countries consume seaweed infrequently, even when they express positive views toward it [39,40,41]. A survey of Australian consumers found that while 62% indicated they would likely eat seaweed in the next 12 months, only 37% reported consuming it at least once per month in the previous year [39]. Similarly, Palmieri and Forleo [42] surveyed Italian consumers and found that although 57% had consumed seaweed previously, 76% expressed willingness to consume it in the future, again suggesting a gap between past behavior and future intention. This pattern was further supported by Pickering et al. [40], whose survey of UK residents revealed low current consumption, but also moderate future interest. In contrast, Japanese respondents surveyed in the same study reported substantially higher current consumption and stronger intent to consume seaweed in the future, highlighting the influence of cultural context on consumption patterns.

Evidence regarding Western consumers’ willingness to incorporate seaweed into their diets has been mixed [43,44]. Moss and McSweeney [44] found that consumers reported higher purchase intent for seaweed presented as a dried standalone ingredient or incorporated into bread, but were less receptive when it was added to more novel food formats such as yogurt or sausage. Chapman et al. [43] evaluated consumer responses to fish cakes with and without 5% w/w seaweed and found that 42% of participants preferred the seaweed-containing product, while 22% expressed no preference between the two. Taken together, these findings suggest that the food matrix into which seaweed is incorporated plays an important role in shaping consumer acceptance. The findings of the present study align with this body of literature and further underscore the need for continued research to support the successful integration of seaweed as a mainstream ingredient within Western food systems.

### 3.2. Consumer Acceptability

Mean hedonic scores for each of the five samples are presented in Table 2 (a spider diagram is also included as Supplementary Material S2). With respect to appearance, the unfermented, AO, and PP samples received significantly lower liking scores than the ST sample (*p* < 0.05). The control received significantly higher flavour liking scores than all other samples (*p* < 0.05), a result that was anticipated given the well-documented consumer preference for chocolate [45]. Among the seaweed-containing samples, the AO sample received significantly higher liking of flavour scores than the unfermented sample (*p* < 0.05). This finding is consistent with previous research demonstrating that AO fermentation can enhance the flavour properties of other seaweed varieties, including sea tangle [10] and purple giant [46], as well as nori sauce produced from *Pyropia yezoensis* [47]. More broadly, fermentation has been identified as a promising strategy for improving the flavour profile of seaweed-containing products [48].

In contrast, the PP and ST samples did not receive significantly higher liking of flavour scores than the unfermented sample (*p* > 0.05). Although both cultures have been proposed as suitable candidates for seaweed fermentation [49], neither produced sufficient changes in sensory characteristics to meaningfully influence consumer flavour liking in this study. PP fermentation has been shown to improve the odour of salted kelp; however, that evaluation was conducted using a trained panel rather than a consumer panel, and salted kelp is a traditional food in China [50], where that study was conducted. The present study differs in both panel composition and food matrix, as seaweed was incorporated into chocolate rather than a culturally familiar product. ST has similarly been associated with improved sensory properties of seaweed when incorporated into yogurt [51,52], though ST is also a standard culture used in yogurt production regardless of seaweed inclusion [53]. To the authors’ knowledge, the present study is the first to use ST for seaweed fermentation prior to incorporation into chocolate. Given that neither PP nor ST produced increased liking of flavour scores significantly different from the unfermented control, AO may represent the most suitable fermentation culture for seaweed destined for inclusion in chocolate.

Liking of texture scores for the control were significantly higher than those of all seaweed-containing samples (*p* < 0.05). This may partially reflect the halo effect, whereby strong liking for one sensory attribute (in this case, flavour) leads consumers to report higher liking across other attributes as well, even when those attributes are theoretically equivalent across products [54]. Additionally, previous research has demonstrated that the incorporation of ground seaweed into food products can reduce textural liking in proportion to the quantity added [55]. The inclusion of finely ground seaweed in the present study may also have disrupted the smooth, creamy mouthfeel that consumers typically associate with chocolate [56], contributing to reduced textural liking across seaweed-containing samples. Notably, the AO, PP, and ST samples all received significantly higher texture liking scores than the unfermented sample (*p* < 0.05), suggesting that fermentation, regardless of culture type, may partially mitigate the negative textural effects of seaweed addition.

With respect to overall liking, the control was rated significantly higher than all seaweed-containing samples, while the AO sample was rated significantly higher than both the unfermented and ST samples (*p* < 0.05). While a mean overall liking score of 6.6 for the AO sample exceeds the threshold of ≥6.0 for acceptable consumer liking of novel food products, however, commercial viability encompasses more than hedonic liking alone. Purchase intent, perceived health benefit, and product familiarity are all known to influence whether laboratory acceptability translates into actual consumer purchasing behaviour, and further studies need to be conducted. Past consumer studies examining the acceptability of seaweed-enriched chocolate remain limited. Salgado et al. [26] evaluated three seaweed–chocolate combinations and found that while hedonic scores were moderate (mean scores ≥ 6 on a nine-point scale across appearance, colour, flavour, texture, and overall liking), purchase intent remained low across all three products (milk-kombu = 3.8; ruby-nori = 3.1; white-sea lettuce = 2.6). This suggests that even when consumers are open to novel ingredients in confectionery, familiarity may take precedence when it comes to comfort foods [57]. Stefani et al. [58] evaluated the sensory properties of milk chocolate fortified with 5% *Eucheuma cottonii* seaweed flour and found non-significant increases in hedonic liking for appearance, aroma, and taste, alongside a non-significant reduction in texture liking. Although these results do not closely align with those of the present study, with the exception of reduced texture liking, they suggest that the choice of seaweed species may substantially influence the sensory outcome of seaweed-enriched chocolate.

**Figure 1:**
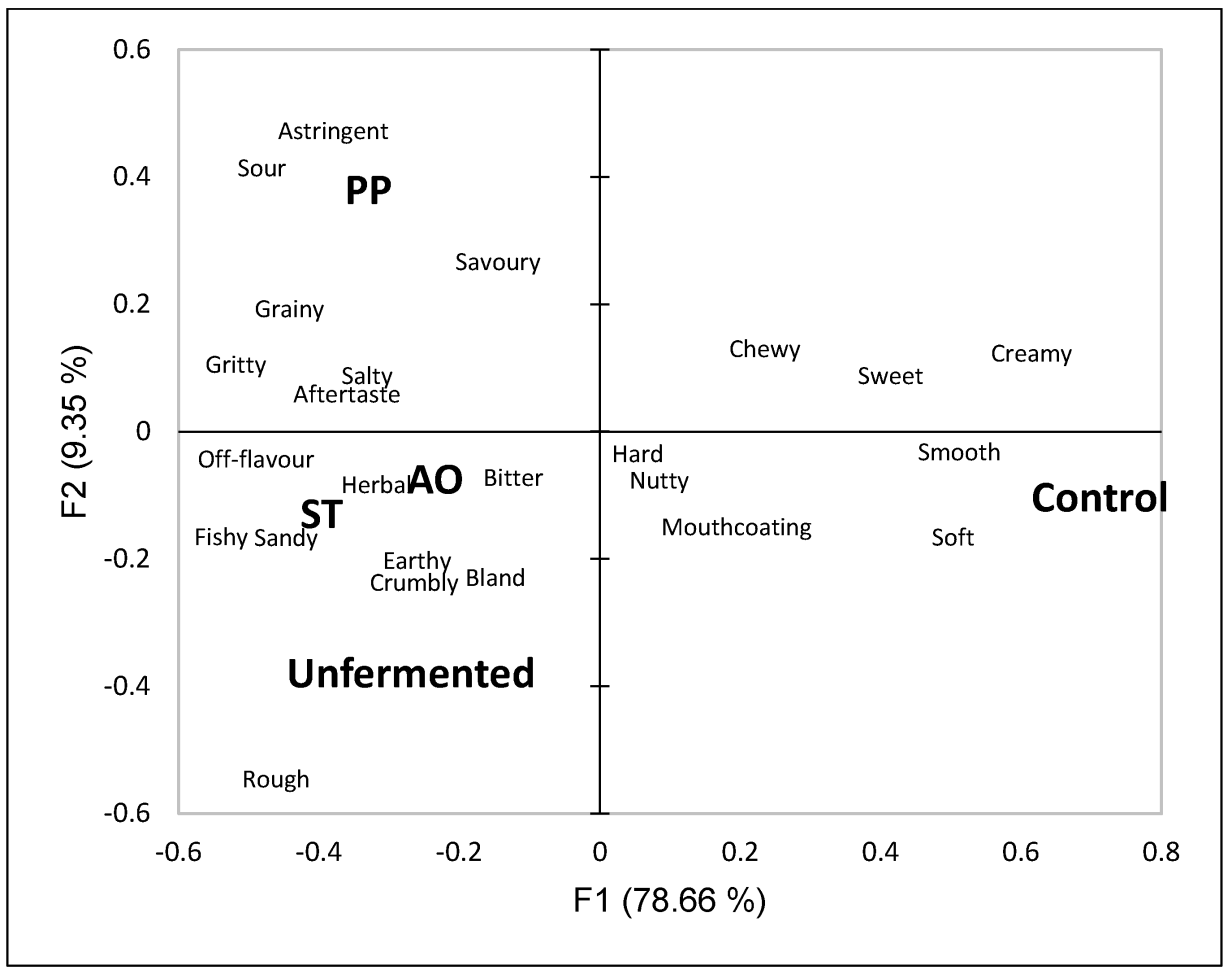
Biplot representation of the sample and sensory properties on the first two dimensions of the correspondence analysis.

Correspondence analysis (CA) of the CATA data (Figure 1) revealed that the first two dimensions accounted for 88.01% of total variation (78.66% and 9.35%, respectively). The first dimension primarily separated the control from the seaweed-containing samples, while the second dimension differentiated among the seaweed-containing samples themselves.

The control was characterized by attributes including “smooth”, “soft”, “creamy”, “sweet”, “mouthcoating”, “hard”, “chewy”, and “nutty”. These descriptors are consistent with established sensory profiles of dark chocolate. Perceived creaminess in chocolate is typically associated with dairy-derived flavour notes and textural attributes such as mouthcoating and viscosity [59] and has been shown to correlate positively with sweetness and negatively with bitterness [60]. Sweetness and texture have also been identified as the primary drivers of overall liking in consumer evaluations of chocolate, exerting greater influence than odour, bitterness, or acidity [61]. Bitterness further influences chocolate preference, with consumers tending to favour dark chocolate varieties perceived as less bitter and sweeter [62]. Nuttiness, which is more prominent in dark than milk chocolate [35], was also associated with the control. Collectively, the sensory profile attributed to the control by participants in this study is consistent with that reported in the broader literature.

The PP sample was characterized by “astringent”, “sour”, “savoury”, “grainy”, “salty”, “gritty”, and “aftertaste”. Fermentation with *Pediococcus* strains has previously been associated with elevated saltiness relative to *Lactobacillus* strains, though with comparatively lower acidity [63], and reduced sourness has also been reported following *Pediococcus* fermentation, albeit non-significantly [64]. Conversely, fermentation of mulberry leaf powder with *P. pentosaceus* has been shown to reduce pH through increases in lactic, acetic, citric, tartaric, and pyruvic acid concentrations [65]. *P. pentosaceus* has also been shown to reduce fishy off-flavours in raw seaweed [66]; however, the authors did not assess the fermentation of cooked seaweed due to the heat sensitivity of the strain. In the present study, cooked seaweed was cooled prior to inoculation, thereby avoiding thermal inactivation of the culture. Despite this, the PP sample remained associated with astringency, sourness, graininess, grittiness, and aftertaste, which are attributes previously linked to reduced liking of both seaweed and seaweed-enriched chocolate [26].

The ST, AO, and unfermented samples clustered together and were characterized by “earthy”, “crumbly”, “bland”, “herbal”, “bitter”, “off-flavour”, “sandy”, “fishy”, and “rough”. Earthy and mushroom-like off-flavours in ST-fermented products have been linked to the accumulation of the volatile organic compound 1-octen-3-ol [67]. ST has a limited capacity to ferment complex polysaccharides [68], which likely restricted its ability to break down seaweed carbohydrate chains relative to PP. AO fermentation of red seaweed (*Kappaphychus alvarezii*) has been shown to increase amino acids associated with bitter and umami taste while reducing those contributing to sweetness [46]. The influence of AO on sensory outcomes is further strain-dependent; Murayama et al. [69] demonstrated that 120-day fermentation of *Pyropia yezoensis* with different koji preparations produced significantly different intensities of bitterness, saltiness, and astringency depending on the strain used, underscoring the importance of precise strain characterization in fermentation studies.

Across all four seaweed-containing samples, off-flavours exerted a substantial negative influence on acceptability. Off-flavours have been shown to significantly reduce consumer acceptance of both plain chocolate and chocolate enriched with additional ingredients [70,71]. Fishy and oceanic off-flavours are a well-documented challenge in seaweed-enriched foods more broadly: seaweed incorporation into pasta has been shown to induce saltiness and reduce liking for appearance, mouthfeel, and overall quality at higher concentrations [72]; fish burgers containing seaweed exhibited varying intensities of fish and algae flavour modulated by seaweed concentration [73]; and seaweed snack development has revealed significant correlations between seaweed concentration and consumer perception of fishy aroma, fishy flavour, seaweed flavour, saltiness, and lingering taste [74].

**Figure 2:**
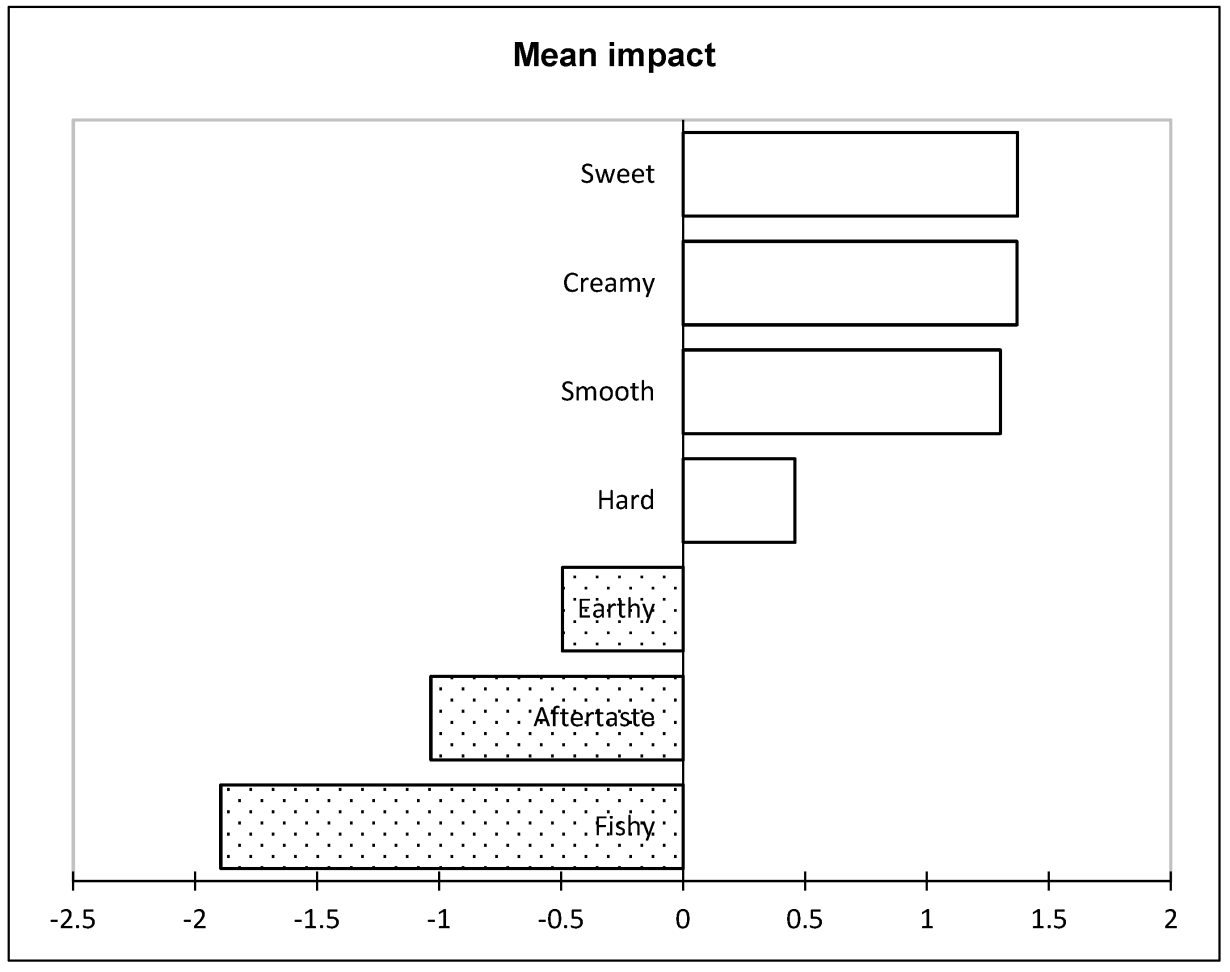
Penalty-lift analysis based on the sensory properties and overall liking identified by the participants.

Penalty-lift analysis (Figure 2) identified “sweet”, “creamy”, “smooth”, and “hard” as the attributes with the greatest positive impact on overall liking, all of which were associated with the control. These findings align with prior research identifying these attributes as key drivers of chocolate acceptability [45,75]. Conversely, “fishy”, “aftertaste”, and “earthy” exerted the greatest negative impact on liking, consistent with sensory profiles previously attributed to seaweed-containing products [49,76,77]. Overall, seaweed addition reduced consumer liking of the chocolate and introduced notable off-flavours across all enriched samples.

A past study by Salgado et al. [26] evaluated three seaweed–chocolate combinations including milk chocolate with kombu, ruby chocolate with nori, and white chocolate with sea lettuce, using binary logistic regression models with large consumer samples (*n* = 104–139 per sample). Their sensory characterization revealed that each seaweed variety imparted distinct attributes to the chocolate; kombu contributed saltiness, nori contributed astringency and a dehydrated fruit note, and sea lettuce was associated with green tea/matcha, marine, and vegetable flavour notes. These findings are broadly consistent with those of the present study, in which seaweed-containing samples were characterized by off-flavours, fishy, earthy, and savoury descriptors that distinguished them markedly from the control. Notably, Salgado et al. [26] reported moderate hedonic scores (mean ≥ 6 across all attributes) but low purchase intent across all three products, suggesting that even when consumers are not strongly averse to seaweed in chocolate, familiarity and comfort food expectations remain a barrier to purchase. This pattern aligns with the reduced overall liking scores observed in the present study for all seaweed-containing samples relative to the control. A key distinction between the two studies is that Salgado et al. [26] incorporated whole or minimally processed seaweeds directly into chocolate, whereas the present study first subjected the seaweed to fermentation prior to incorporation.

### 3.3. Emotional Response

**Figure 3:**
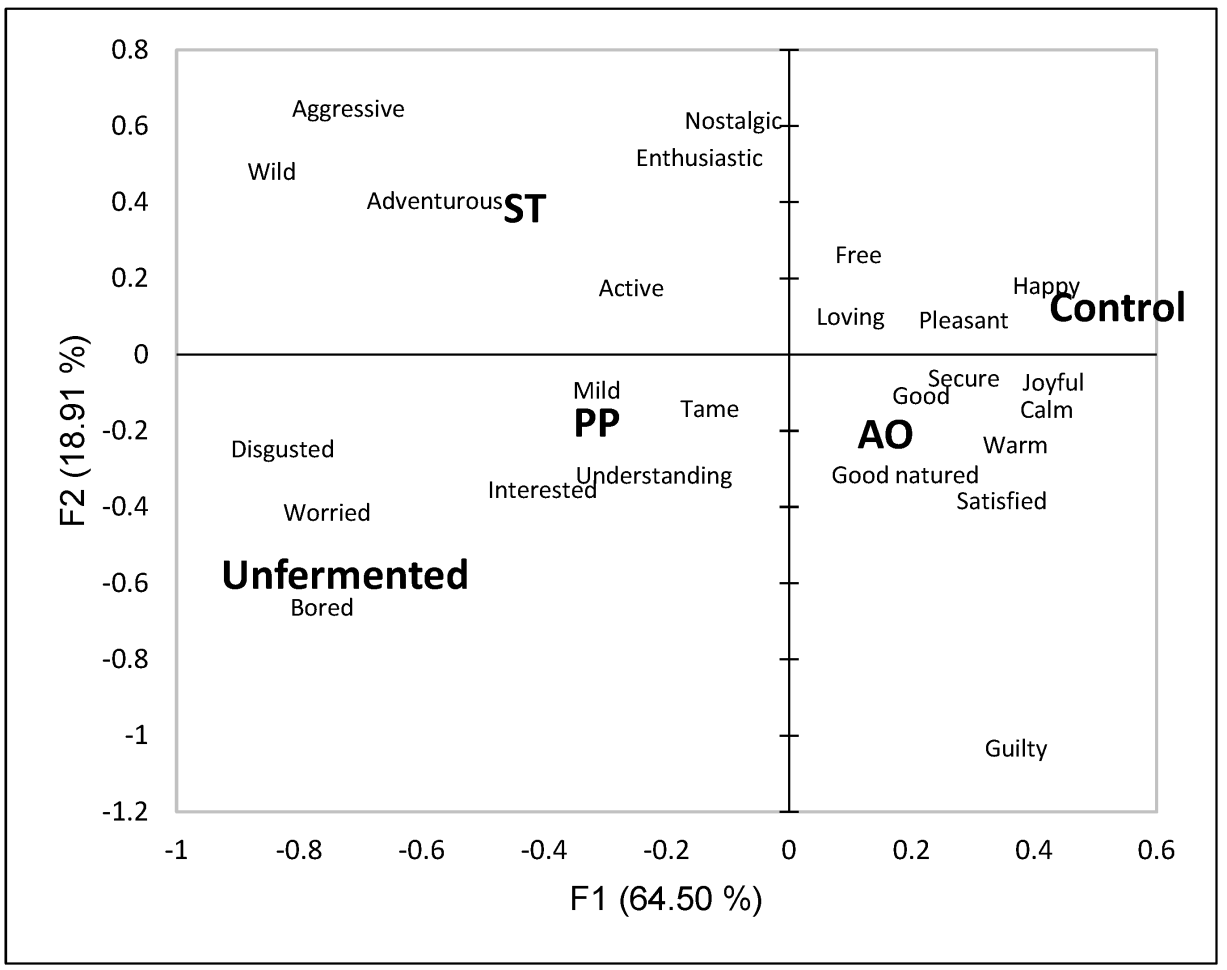
Biplot representation of the samples and the emotional responses on the first two dimensions of the correspondence analysis.

Correspondence analysis of the EsSense25 CATA data (Figure 3) revealed that the first two dimensions accounted for 83.41% of total variation (64.50% and 18.91%, respectively). The 25 terms comprising the EsSense25 questionnaire are classified as positive (17 terms), negative (3 terms), or unclassified (5 terms), and are derived from the original 39-term EsSense Profile^®^ with classifications unchanged from their original context [78].

The control sample was associated with four positive terms: “happy”, “pleasant”, “loving”, and “free”. The AO sample was associated with seven positive terms, “good-natured”, “good”, “secure”, “joyful”, “calm”, “warm”, and “satisfied”, along with one unclassified term, “guilty”. The ST sample was associated with four positive terms (“adventurous”, “active”, “enthusiastic”, and “nostalgic”) and two unclassified terms (“wild” and “aggressive”). The PP sample was associated with two positive terms (“understanding” and “interested”) and two unclassified terms (“mild” and “tame”). Notably, the unfermented sample was exclusively associated with the three negative terms: “bored”, “worried”, and “disgusted”.

Positive emotions have been broadly correlated with higher liking scores, while the three negative terms, “bored”, “disgusted”, and “worried”, are associated with reduced liking [24]. However, these relationships do not always hold in experimental contexts. A study comparing the EsSense Profile to the EmoSensio profile among Italian consumers evaluating chocolate found that “aggressive”, “guilty”, “worried”, and “nostalgic” could each correlate negatively with overall liking [79]. That study also found that “eager” strongly correlated with disliking in one trial, which the authors attributed to the more negative connotation carried by its Italian equivalent (“avido”), underscoring the importance of careful linguistic consideration when designing emotion-based questionnaires for use across different cultural contexts [79]. In a study of frequent chocolate consumers in Belgium and Hungary, De Pelsmaeker et al. [80] further demonstrated that anticipated emotional responses can positively or negatively influence liking when a product fails to meet sensory expectations.

**Figure 4:**
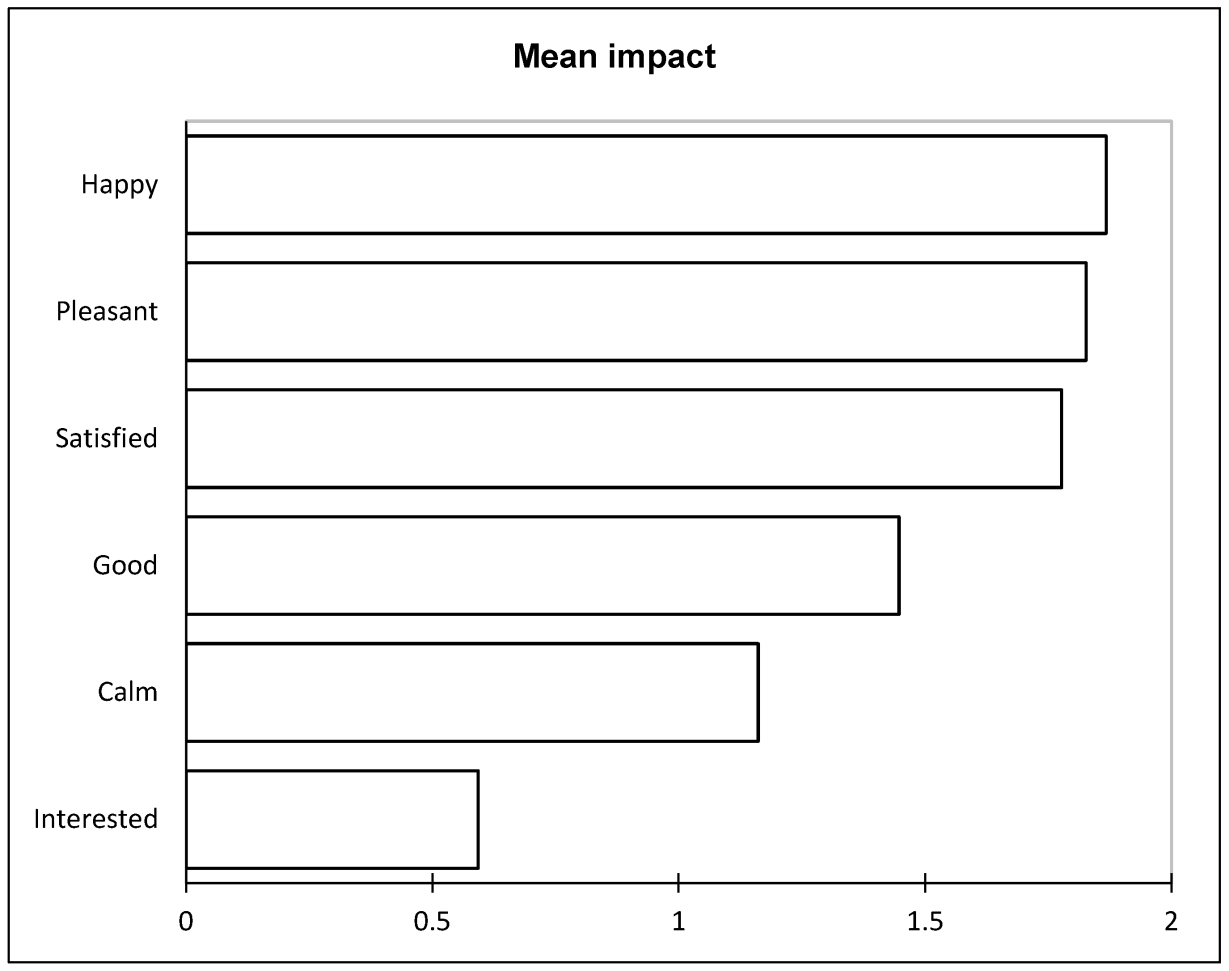
Penalty lift analysis of the emotional responses and overall liking based on the participants’ perception.

Penalty-lift analysis of the emotional response data (Figure 4) identified “happy”, “pleasant”, “satisfied”, “good”, “calm”, and “interested” as the terms with the greatest positive impact on overall liking, all of which are classified as positive emotions. These findings are consistent with prior research on emotional responses to chocolate [81,82] and with the broader literature linking positive emotional responses to increased consumer liking [83,84].

### 3.4. Limitations and Future Directions

This study examined consumer perception of dark chocolate enriched with a single seaweed variety, fermented using three different cultures or left unfermented. Several avenues exist for future research to build upon these findings. The fermentation cultures selected in this study were chosen based on the prior literature; future work could investigate alternative cultures, different fermentation processes, seaweed species, or chocolate matrices, including milk versus dark chocolate or varying chocolate-to-seaweed ratios, to more comprehensively characterize consumer acceptance of seaweed-enriched confectionery. Furthermore, studies could explore the potential of fermented seaweed used in conjunction with complementary flavour ingredients, such as sea salt, orange, or mint, which may help mask marine and off-flavour attributes. Volatile compound profiling of fermented seaweed–chocolate products would also complement the sensory data generated here and facilitate comparison with existing literature. Future studies would benefit from incorporating screening questionnaires to assess the influence of prior attitudes toward seaweed and food neophobia on hedonic scores, as these factors may confound sensory-driven liking responses. The particle size of the seaweed powder used in this study (sieved to 130 µm) likely contributed to the sandy and gritty textural perceptions reported by consumers across seaweed-containing samples. It is well-established that particles above approximately 30 µm are perceptible in chocolate and generate negative textural attributes [85]. Future work should investigate finer milling techniques to reduce seaweed particle size below this threshold, which may improve textural liking and overall consumer acceptance of seaweed-enriched chocolate products. Also, the participants predominantly identified as females and future studies should aim to recruit a more balanced gender distribution; however, that may reflect who is interested in foods containing seaweed, as all participants were recruited based on willingness to consume seaweed. Finally, as this study was conducted with residents of Atlantic Canada, cross-cultural research would be valuable in determining whether geographic location and cultural familiarity with seaweed influence consumer perception of these products.

## 4. Conclusions

This study evaluated the sensory impact of fermenting *Porphyra umbilicalis* (laver) with *Streptococcus thermophilus* (ST), *Pediococcus pentosaceus* (PP), and *Aspergillus oryzae* (AO) before incorporation into dark chocolate, using hedonic scales, CATA, and the EsSense25 emotional profiling tool. All seaweed additions (fermented or unfermented) significantly reduced consumer liking relative to the control, which was associated exclusively with positive sensory attributes and emotions. However, AO-fermented seaweed achieved the highest flavour and overall liking scores among seaweed-containing samples, demonstrating the superiority of fungal over bacterial fermentation in mitigating undesirable sensory characteristics. Emotional responses mirrored liking scores, reinforcing the link between emotional response and consumer acceptance in chocolate products.

These findings provide a foundation for strain selection in future seaweed fermentation research and highlight the need to investigate food matrices, such as savoury products or baked goods, where the sensory attributes introduced by fermentation may be better received. Future studies should investigate lower incorporation levels of fermented laver (e.g., 3–4% *w*/*w*) to determine whether a reduced concentration can improve consumer acceptability and minimise the negative sensory attributes associated with the 6% *w*/*w* level used in the present study. Future work should also examine the physicochemical and structural changes underlying these sensory outcomes. Fermentation remains a promising strategy for improving the palatability and broader integration of seaweed into Western diets.

## Figures and Tables

**Table 1:** Participants’ beliefs about seaweeds.

Statement	Mean ± Standard Deviation
Foods that contain seaweed are healthy.	4.0 ^1,2^ ± 0.7
Seaweed is a good source of antioxidants.	4.1 ± 0.6
Seaweed is a good source of vitamins.	4.2 ± 0.7
Seaweed is a good source of omega-3 fatty acids.	3.7 ± 0.9
Foods containing seaweed are disgusting.	2.1 ± 1.0
Seaweed can be part of a healthy diet.	4.3 ± 0.7

^1^ Data input on a five-point scale where 1 = Strongly Disagree and 5 = Strongly Agree. ^2^ *n* = 83.

**Table 2:** Consumer mean liking scores (±standard deviation) for appearance, flavour, texture, and overall liking of the samples.

Sample	Appearance	Flavour	Texture	Overall Liking
Control	7.5 ab^1,2,3^ ± 1.2	7.8 a ± 0.9	7.9 a ± 0.8	7.8 a ± 0.9
Unfermented	7.3 b ± 1.2	5.7 c ± 1.8	5.7 c ± 1.8	5.7 c ± 1.8
AO	7.3 b ± 1.3	6.5 b ± 1.7	6.6 b ± 1.5	6.6 b ± 1.5
PP	7.3 b ± 1.3	6.2 bc ± 1.8	6.7 b ± 1.5	6.1 bc ± 1.8
ST	7.6 a ± 1.1	6.0 bc ± 2.0	6.8 b ± 1.4	6.0 c ± 1.9

^1^ Data input on a nine-point hedonic scale where 1 = Dislike Extremely and 9 = Like Extremely. ^2^ Means in the same column with the same letter are not significantly different (*p* < 0.05). ^3^ *n* = 83.

## Data Availability

The data presented in this study are available on request from the corresponding author due to ethical reasons.

## Supplementary Materials

The following supporting information can be downloaded at https://www.mdpi.com/article/10.3390/foods15122047/s1, Supplementary Material S1—Sensory Questionnaire (Presented using Compusense Software); Supplementary Material S2—Spider diagram of consumers’ mean liking scores.
