# Supplementary material for: Enhancing Dark Chocolate with Fermented Laver (Porphyra umbilicalis): Effects on Sensory Characteristics and Consumer Acceptance
Source: Foods. 2026 Jun 6;15(12):2047. doi: 10.3390/foods15122047 (PMC13298137; doi:10.3390/foods15122047)

Supplementary Material S1- Sensory Questionnaire (Presented using Compusense Software)

Please look at sample BLINDING CODE.
How much do you like the appearance of sample BLINDING CODE.

☐ ☐ ☐ ☐ ☐ ☐ ☐ ☐ ☐

Dislike Extremely — Like Extremely

Please take a bite of sample BLINDING CODE.

How much do you like the flavour of sample BLINDING CODE.

☐ ☐ ☐ ☐ ☐ ☐ ☐ ☐ ☐

Dislike Extremely — Like Extremely

How much do you like the texture of sample BLINDING CODE.

☐ ☐ ☐ ☐ ☐ ☐ ☐ ☐ ☐

Dislike Extremely — Like Extremely

Overall, how much do you like sample BLINDING CODE.

☐ ☐ ☐ ☐ ☐ ☐ ☐ ☐ ☐

Dislike Extremely — Like Extremely

Please click all of the attributes that you perceive in sample BLINDING CODE.

Sweet	Bitter	Astringent
Bland	Nutty	Grainy
Rough	Chewy	Soft
Hard	Crumbly	Herbal
Salty	Savoury	Aftertaste

Fishy	Sour	Sandy
Smooth	Gritty	Creamy
Earthy	Mouthcoating	Off-flavour

How does sample BLINDING CODE make you feel? Click all that apply.

Happy	Joyful	Good
Interested	Pleasant	Good natured
Secure	Satisfied	Free
Understanding	Enthusiastic	Loving
Adventurous	Aggressive	Wild
Active	Nostalgic	Warm
Mild	Calm	Tame
Bored	Disgusted	Guilty
Worried		

Supplementary Material S2- Spider diagram of consumers' mean liking scores

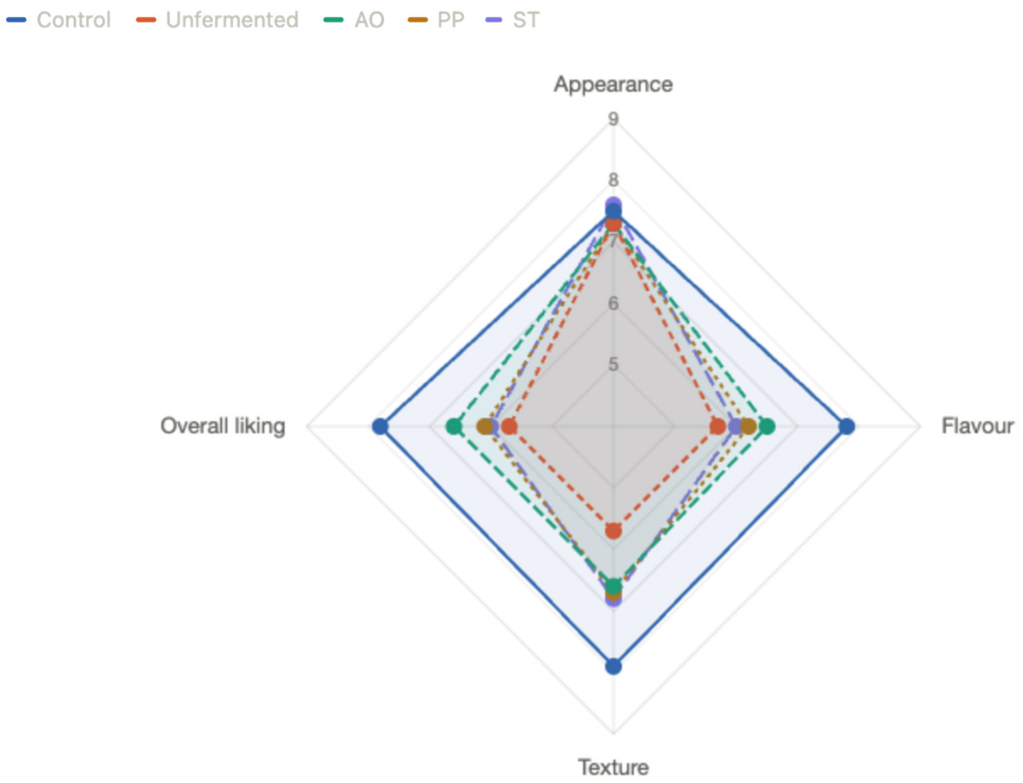

Supplement: Supplementary file 1 [file foods-15-02047-s001.zip › foods-4305534-supplementary.pdf]
